# Minimal Residual Disease Assessment in Multiple Myeloma Patients: Minimal Disease With Maximal Implications

**DOI:** 10.3389/fonc.2021.801851

**Published:** 2022-01-26

**Authors:** Charalampos Charalampous, Taxiarchis Kourelis

**Affiliations:** Division of Hematology, Mayo Clinic, Rochester, MN, United States

**Keywords:** multiple myeloma, minimal residual disease, liquid biopsy, NGS, NGF

## Abstract

Multiple Myeloma (MM), the second most common hematologic malignancy, has been the target of many therapeutic advances over the past two decades. The introduction of novel agents, such as proteasome inhibitors, immunomodulatory drugs, and monoclonal antibodies, along with autologous hematopoietic stem cell transplantation (ASCT) in the current standard of care, has increased the median survival of myeloma patients significantly. Nevertheless, a curative treatment option continues to elude us, and MM remains an incurable disease, with patients relapsing even after achieving deep conventionally defined responses, underscoring the need for the development of sensitive methods that will allow for proper identification and management of the patients with a higher probability of relapse. Accurate detection of Minimal Residual Disease (MRD) from a bone marrow biopsy represents a relatively new approach of evaluating response to treatment with data showing clear benefit from obtaining MRD(-) status at any point of the disease course. As life expectancy for patients with MM continues to increase and deep responses are starting to become the norm, establishing and refining the role of MRD in the disease course is more relevant than ever. This review examines the different methods used to detect MRD and discusses future considerations regarding the implementation in day-to-day clinical practice and as a prospective primary endpoint for clinical trials.

## Introduction

In recent years, with the advent of new therapeutic regimens and monoclonal antibodies, the landscape of treatment options for Multiple Myeloma (MM) has substantially changed, leading to significantly increased complete response (CR) rates and prolonged survival (1, 2). As new drugs and combinations of different classes are quickly becoming the standard of care, accurate quantification of disease response has become essential for the risk stratification and management of patients with the highest relapse risk after therapy.

Patients achieving a deep response, defined as CR or higher, have prolonged progression-free survival and overall survival compared to non-CR patients, a finding that multiple studies have confirmed (3–5). As a result, an early goal of therapy is to attain deep remissions, and nearly 80% of patients are achieving near-complete responses with modern induction therapy (6, 7). This is also true for older, transplant-ineligible patients (8). However, even these patients are consistently relapsing, indicating the urgent need to incorporate more sensitive methods for response assessment (4). It is now becoming clear that minimal residual disease (MRD) negativity is a robust prognostic indicator in MM, even in patients with conventional CR. Indeed, patients with MRD(-) CR status have enjoyed prolonged disease-free periods compared to those in MRD(+) CR, and on many occasions, irrespective of the presence of high-risk disease features (9). For this reason, the latest 2016 International Myeloma Working Group has introduced new response criteria (10), with the addition of MRD in disease assessment both in the bone marrow (BM) and in extramedullary sites (through imaging).

With additional validation, MRD has the potential to serve as a surrogate marker of clinically relevant endpoints such as OS and can be reported much sooner, therefore accelerating drug development. Additional studies also focus on response-adapted approaches based on MRD, such as escalating therapy in MRD(+) patients or de-escalating in MRD(-) patients. As a result, standardized and accessible MRD assessment across the disease spectrum of Myeloma (newly diagnosed to heavily pre-treated disease) could become a useful tool in drug development and clinical management of patients.

In this review, we discuss the different methods currently used for MRD assessment, along with their respective strengths and weaknesses, the role of imaging in complementing the evaluation, especially for extramedullary disease patients, and what we know about the true prognostic impact of MRD at different time points in the disease course. Also, we assess the current knowledge regarding the utilization of MRD as a driver of clinical decisions in the future. Finally, we discuss existing limitations in the use of MRD in clinical practice.

## Bone Marrow Assessment Methods

The two currently validated methods utilized for the detection of MRD in the BM are Next Generation Flow cytometry (NGF), which uses distinctive cell surface and cytoplasmic markers for clonal plasma cell detection, and Next Generation Sequencing (NGS), using specific V(D)J rearrangements for clonality identification (10). The IMWG consensus defined the MRD negative state as the absence of phenotypically aberrant clonal plasma cells, assessed by NGF on BM aspirates, using the EuroFlow standard operation procedure (or a validated equivalent method) with a minimum sensitivity of 1 in 10^5^ nucleated cells or higher. The same level of sensitivity was suggested for the NGS method. The sensitivity threshold, albeit prognostically relevant, was primarily based on the available efficacy data and reliable technological detection limits at the time. In the past five years, significant advances have been made in optimizing the existing means of detecting MRD in the BM of MM patients. Since then, both methods are adjusted to detect clonal plasma cells with a sensitivity of 10^-6^ or higher (11).

### NGF (Next Generation Flow Cytometry)

Immunophenotyping and predominantly multicolor flow cytometry (MCF) was utilized early on to monitor MRD in MM, primarily due to its availability across institutions and ease of obtaining and interpreting its results (6, 12, 13). Conventional 4-8 color flow cytometry methods can detect aberrant plasma cells with a sensitivity of 10^-4^. MCF recognizes abnormal cells based on distinct cell surface and cytoplasmic malignant markers, with the most commonly used ones being CD138, CD38, CD45, CD56, CD117, CD19, and κ,λ light chains (14). Aside from these, additional markers are currently being investigated. This prospect will aid in the identification of malignant clones, particularly after treatment with anti-CD38 monoclonal antibodies, which can decrease the level of CD38 expression on malignant plasma cells (15). The prognostic value of flow-cytometry based methods was demonstrated in the IFM2009 study comparing lenalidomide-bortezomib-dexamethasone (VRd) with consolidative *vs.* salvage autologous stem transplant(ASCT) therapy. In that study, patients with double negativity with the MCF (sensitivity of 1 cell in 10^4^) and NGS(sensitivity of 1 cell in 10^6^) had superior PFS than those who tested negative only for the MCF method. These findings also highlight the importance of using MRD detection methods with a higher sensitivity (16).

Despite the well-established benefits of the MCF in MRD assessment, the lack of consistent antibody panels and the relatively low sensitivity, especially when contrasted with the other methods being used, created a consistency reporting problem. The Euroflow consortium developed a next-generation, 8 color, 2 tube flow cytometer with standardized antibodies targeting specific epitopes and sensitivity of 2x10^-6^, provided that an adequate BM sample has been collected (approximately 10 million cells) and promptly processed (within 48 hours) (15). The first tube contains the antibodies of the most common cell surface markers found in abnormal myeloma cells, whereas the second contains 6 cross-references and the cytoplasmic markers, namely κ,λ lights chains (**Table 1**). The use of the Euroflow tubes has allowed for uniform, accurate, and reproducible result reporting for MRD detection, and this approach can reclassify up to one-quarter of patients that were thought to be MRD(-) with less sensitive methods and thus provides incremental prognostic information. Even though similarly sensitive MCF methods are available (17), the IMWG has incorporated Euroflow as the reference for establishing the MRD negative status when utilizing flow cytometry.

**Table 1:** Euroflow antibody markers used in the 2 *tube* approach.

Euroflow MFC antibodies	
**Tube 1**	**Tube 2**
CD138	CD138
CD38	CD38
CD19(97%)	CD19
CD45(89%)	CD45
CD81(86%)	CyIgκ
CD56(86%)	CD56
CD27(71%)	CD27
CD117(60%)	CyIgλ

1. CD38, CD 138 Were used to detect plasma cell population in the sample. 2. Percentages correspond to the consistency of discrimination between normal vs. aberrant plasma cells. 3. CyIgλ, CyIgκ cytoplasmic markers were used to identify clonality.

The prognostic value of Euroflow has been validated in the PETHEMA/GEM2012MENOS65 trial, which showed that patients with undetectable MRD had an 82% reduction in the risk of progression or death (hazard ratio, 0.18; 95% CI, 0.11 to 0.30; P<.001) and an 88% reduction in the risk of death (hazard ratio, 0.12; 95% CI, 0.05 to 0.29; P,.001), compared to those who tested positive. Notably, reaching undetectable MRD negated poor prognostic features at diagnosis, including high-risk cytogenetics and initial R-ISS classification. Specifically, with a median follow-up of 40 months, disease progression occurred in 14 patients (7%) with undetectable MRD versus 101 patients (40%) with persistent MRD after consolidation (P <.001) (18).

Apart from Euroflow, a recently developed US version is based on a 10-color single tube panel with a sensitivity of 6 tumor cells in 1,000,000, provided 3 million cells can be assayed. The MSKCC single-tube method combines all surface and cytoplasmic staining into a single tube following bulk lysis to further streamline the process and provide similar sensitivity to Euroflow (17).

Although MFC, and especially the Euroflow technique, has solved many issues regarding the applicability and generalizability of the results produced, some obstacles regarding the method’s feasibility need to be addressed. Firstly, the blood marrow sample, apart from requiring up to 10,000,000 cells for consistent disease detection, must be rapidly processed for the cells to remain viable (an estimate of 85% viability is required). In addition, the use of targeted antibody therapy towards cell surface markers (e.g., CD38), although in large part tackled by the Euroflow method, may still result in false-negative results. Finally, as with all BM-based approaches, myeloma’s heterogeneous, patchy nature in the marrow makes sampling errors possible. This is also true for the presence of extramedullary disease, with BM biopsies having little to do with disease detection.

### NGS (Next-Generation Sequencing)

Molecular techniques can also reliably be employed to detect MRD since they offer precise disease activity measurements with excellent sensitivity (exceeding 10^-6^ in some cases). These methods exploit the uniqueness of Variable, Diversity, Joining rearrangements (VDJ) recombinations of the CdR3 region in the immunoglobulin heavy chain variable (IGH) gene, specific to each clone for identification and amplification of the sequence of interest (19, 20). Briefly, NGS relies on the Variable, Diversity, Joining rearrangements in the IGH gene in primary lymphoid organs (BM). This generates a wide range of amino acid sequences that recognize different antigens in bacteria, viruses, parasites, etc. The same can be applied for the light chain genes (κ, λ), but because they lack the D segment, the method’s sensitivity regarding accurate detection of the clone is reduced. Using this physiologic process of unique IgH production, NGS detects and amplifies the clone that is malignant at the time of diagnosis through specific primers that are not patient-specific (20). Importantly, when the malignant transformation of the B-cell occurs, the exact VDJ arrangement will be retained in all clone cells while being consistently absent from the normal cells. The only FDA-approved NGS assay for BM detection of MRD is the clonoSEQ^®^ assay (Adaptive Biotechnologies; Seattle, WA) which is validated for analytical sensitivity (21). The development of the NGS method has largely replaced the allele-specific oligonucleotides quantitative polymerase chain reaction (ASO-qPCR). Although very sensitive, the latter is not widely applicable since patient-specific probes that can reliably identify clonal IgH after somatic hypermutation occurred were isolated for 50-60% of patients compared to 90-92% with the NGS method (not patient-specific) (20, 22–24). This demonstrates the general applicability of NGS sequencing without the need for laborious development of patient-specific primers.

When considering assays at similar levels of analytical sensitivity, NGF and NGS correlate highly at a 10^-5^ sensitivity, with >90% of CR patients being MRD(-) with both assays (25, 26). However, there appears to be more discordance for assays with a sensitivity of 10^-6^. Multiple NGS-based studies have validated its prognostic impact. Martinez et al. (27) used sequencing to detect MRD in the BM of 133 patients with MM after achieving very good partial response (VGPR) or better in their treatment. Initial clone identification was successful for 91% of patients. MRD(-) negative patients had a substantially longer time to tumor progression (TTP) (median 80 *vs.* 31 months; *P* <.0001) and overall survival (median not reached *vs.* 81 months; *P* = .02), compared with patients who were MRD(+). Using different detection sensitivity levels, the respective TTP medians were: MRD ≥10^−3^ 27 months, MRD 10^−3^ to 10^−5^ 48 months, and MRD <10^−5^ 80 months (*P* = .003 to.0001). 92% of VGPR patients were MRD(+). Of note, when examining patients who achieved complete response, the TTP remained significantly longer for MRD(-) compared with MRD(+) patients (131 *vs.* 35 months; *P* = .0009). These results also underline the incremental prognostic value of increasing sensitivity thresholds in MRD assays. Another study utilized patients from the Spanish GEM2012 trial, comparing the NGS (LymphoTrack^®^) and NGF (Euroflow) methods for detection of MRD, and NGS was found to be sufficiently concordant with NGF, with only 15 out of the 106 patients studied having contradictory results and 3 of them eventually relapsing. Notably, while the threshold for MRD positivity was defined at 10^-4^ in the study, most cases registered as positive with the NGS method, and negative for NGF were above the 10^-5^ mark, perhaps highlighting the lower sensitivity detection limits of the latter. When evaluating patients that relapsed, highly concordant results were seen (23 were double-positive, and 5 were double-negative, perhaps explained by the extramedullary component of their disease) (28).

It is easy to see why these two methods have prevailed in evaluating MRD in MM patients. Both have very sensitive detection rates with very good concordance in the results and have been adequately standardized for general use, with little user-dependent expertise required in the flow cytometry Euroflow automated software package. In addition, NGF allows for global interpretation of the immune microenvironment of the BM since other cell populations can be assessed simultaneously. Thus it can detect the presence of hemodilution in a given sample by the decreased number of non-plasma cells detected (i.e., mast cells). This critical issue can primarily be addressed by having the first pull of the aspiration assayed for MRD analysis as the subsequent ones may not have the same cell quality. Advantages of the NGS method include that fewer cells are needed to achieve the sensitivity required (approximately 3 million compared to 10 million with NGF) and the ability to freeze and store cells for future analysis. On the other hand, up to 10% of blood marrow samples cannot be analyzed with NGS following the initial identification because the clones undergo somatic hypermutation, an antigen-specific process, making the initial clone unidentifiable for the subsequent BM pulls.

**Table 2** summarizes the different methods used for MRD assessment in the bone marrow along with their respective advantages and disadvantages.

**Table 2:** MRD methods used for bone marrow biopsy assessment (10, 15).

	Allele-specific oligonucleotide qPCR	NGF	NGS
**Applicability**	50-70%	Nearly 100%	≥90% (limited mainly by somatic hypermutation of the originally identified malignant clone)
**Baseline Sample**	Required; Patient specific probes also required	Not required	Required; Patient-specific probes are not required
**Quantity of sample required**	<1 million cells	Up to 10 million for 10^-6^ sensitivity	3 million for 10^-6^ sensitivity
**Sample processing**	Can be delayed; can use both fresh and stored samples	Needs to be processed within 48hs	Can be delayed; can use both fresh and stored samples
**Sample quality control**	No	Yes (highly reproducible detection of hemodilution in each sample)	No
**Sensitivity**	≥1 in 10^-5^	≥1 in10^-5^	≥1 in 10^-5^ (only limited by the number of cells provided by the biopsy)
**Additional information**	No further information available	Ability to evaluate bone marrow microenvironment and hematologic subpopulations (e.g., mast cells)	Information about immunoglobulin gene repertoire of B cells in the studied patient samples
**Turnaround for results**	1-2 weeks. Labor intensive	3-4 hs with automated software available	Can take several days; requires heavy bioinformatic support
**Availability**	Wide	Most hospitals with four-color flow cytometry. Eight or more color flow cytometry requires more experienced centers/laboratories. Many laboratories have adopted the EuroFlow laboratory protocols and use the EuroFlow MRD tubes	So far limited to one company/platform that has FDA approval
**Cost**	Approximately 1500 USD at diagnosis, 500 USD at follow-up	Approximately 500 USD/sample	Approximately 1100 USD/sample

IMWG, International Myeloma working group; PCR, polymerase chain reaction; NGS, next-generation sequencing; NGF, next-generation flow; H, hours.

## Blood-Based Methods

Much as the methods analyzed above can provide valuable prognostic information, they are inherently limited to the BM and thus constrained in terms of monitoring extramedullary disease. Furthermore, patchy-multifocal infiltration of the BM can introduce false-negative results in the biopsy pull if the most disease-heavy spot is missed. To tackle these issues, “liquid biopsy” methods for detection of either residual cells (e.g., circulating malignant plasma cells, cell-free DNA) or trace quantities of M protein are being developed, offering the added advantage of routine testing without the need for invasive procedures (29). Unfortunately, existing NGF or NGS approaches for MRD detection in the BM have suffered from issues of low sensitivity and poor concordance across assays when used in peripheral blood (30, 31).

Many studies have associated high numbers of circulating plasma cells (cPC) at diagnosis with worse outcomes, representing an independent prognostic factor of adverse events (32–34). Molecularly, it is hypothesized that the presence of these cells denotes a distinct biological entity with a more self-sustaining course, reflected by low expression of integrins, subclonal mutations specific to the peripheral cells, and thus low dependence of BM microenvironment (35, 36). When comparing the post-treatment MRD assessment using flow cytometry in the BM and the peripheral blood, Luzalba Sanoja-Flores et al. (30) showed that a significant proportion of MM cases that were BM MRD(+) still had undetectable cPC in paired blood samples (55/137 (40%)), whereas every cPC(+) case in the cohort was also BM MRD+. This suggests that cPC(+) patients could avoid a bone marrow biopsy for ascertaining MRD status, although it would be unusual for these patients to be in a conventional CR. Despite the relative lack of accuracy compared with BM-based methods, the role of cPCs in the context of extramedullary disease or multifocal BM disease is still significant, and further research on the topic will allow for the incorporation into regular clinical practice. Non-conclusive results are also found with the cell-free DNA assessment for MRD status. In 2018, Mazzoti et al. (37) showed a 51% discrepancy between paired BM and peripheral blood samples, with all except one case being positive for BM (by NGS) and negative for PB.

**Figure 1:**
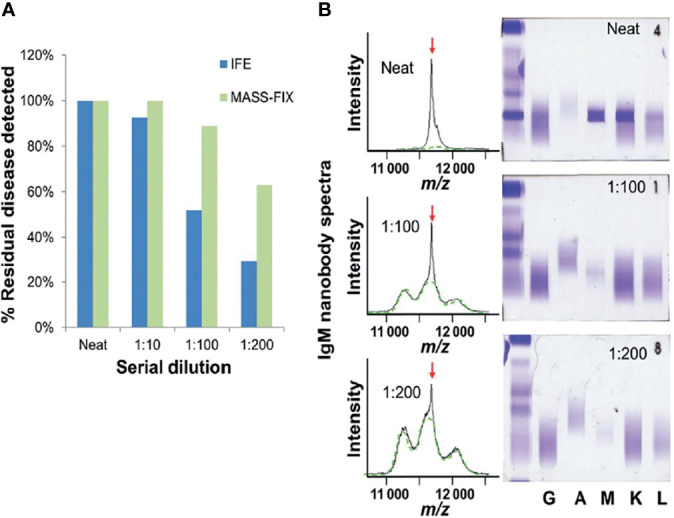
Twenty-seven patients with dysproteinemias representing all possible isotypes (GK, DL, AK, AL, MK, ML, and free kappa and lambda) were analyzed with MASS-FIX (Mayo Clinic’s MALDI-TOF mass spectrometry assay) and immunofixation. The original sample was diluted into normal human serum and serially diluted, as shown in **(A)**. The gels and spectra were randomized and blindly evaluated for the presence of a monoclonal protein by two investigators with a 100% concordance rate. The bar graph in **(B)** demonstrates the superior analytical sensitivity of the mass spectrometry approach over immunofixation. A characteristic example from a monoclonal MK protein is shown on the right. A monoclonal peak is clearly discernible at a 1:200 dilution with MASS FIX but not immunofixation—image courtesy of Dr. David Murray, Director, Mayo Clinic Immunology laboratory.

**Figure 2:**
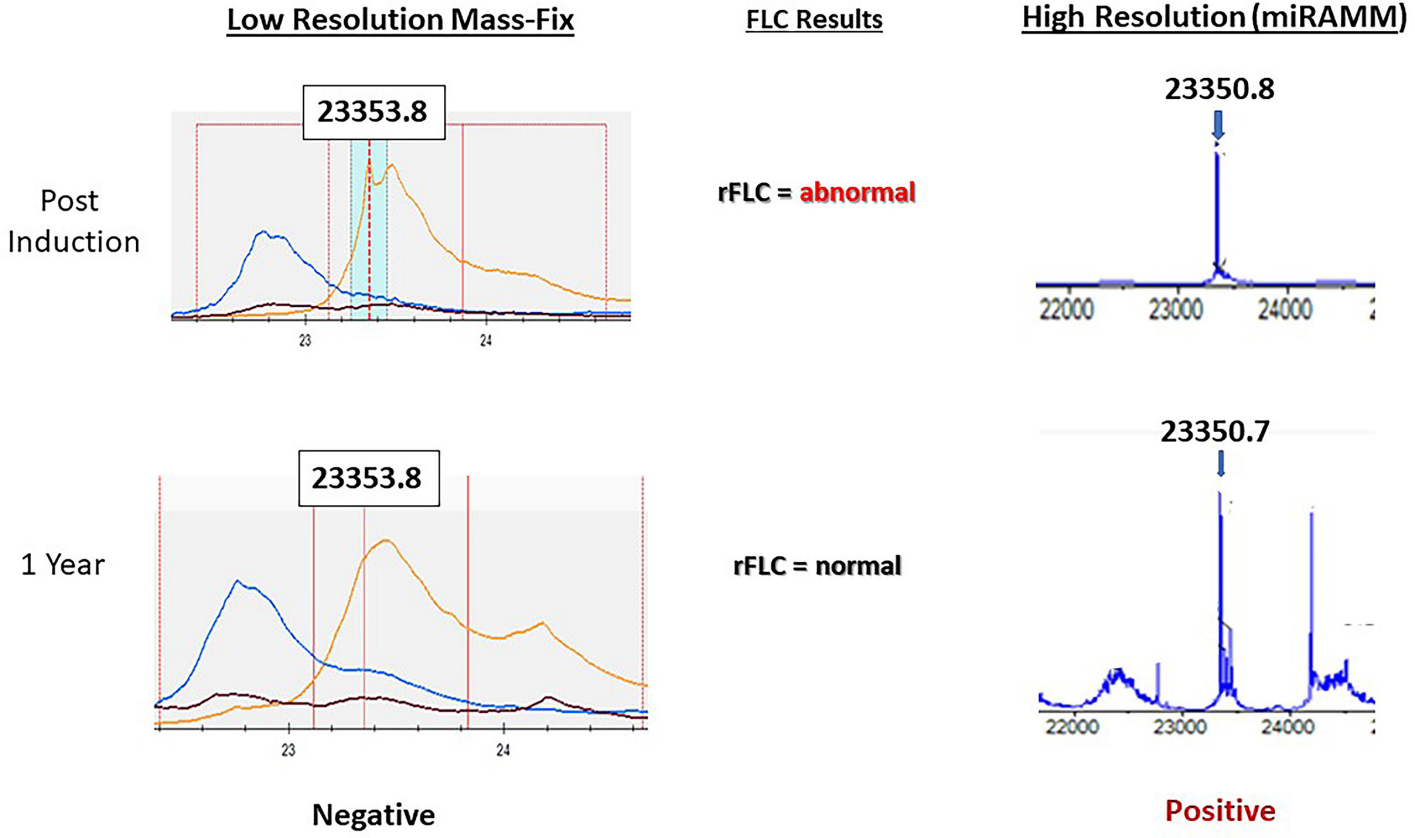
Representative example showcasing the superior sensitivity of the miRAMM approach over MASS-FIX (Mayo Clinic’s MALDI-TOF based approach). A patient with monoclonal kappa multiple myeloma early and one year after induction therapy. After the normalization of the free light chains and the free light chain ratio at one year from diagnosis, MASS FIX became negative, whereas miRAMM remained positive. Bone marrow MRD assessment by multicolor flow was also positive (not shown) at the time. This example suggests that MALDI-TOF based approaches are less likely to be the answer to MRD assessment using peripheral blood, but more sensitive methods are currently being compared to flow cytometry techniques for MRD assessment. Image courtesy of Dr. David Murray, Director, Mayo Clinic Immunology laboratory.

Another modality that can be used for response characterization is M-protein monitoring with mass spectrometry (MS). One MS method is based on isolating intact immunoglobulins from the patient’s serum and then cleaving them into smaller pieces with trypsin digestion, creating distinct amino acid sequences with distinct masses that can be measured and accurately quantified (“bottom-up” approaches). The target for identifying the proteins of interest is the unique sequence of the antigen-binding region, also called the complementarity determining region (CDR) of the Ig. Importantly, this method can identify monoclonal protein with 100 times greater sensitivity than the familiar immunofixation and 200 times when compared to electrophoresis while also distinguishing myeloma-derived proteins from therapeutic monoclonal antibodies (38) (**Figure 1**). This technique, although very promising, has not been studied extensively in comparison with the BM gold standards. Another approach is termed miRAMM (monoclonal Ig rapid accurate mass measurements) and is based on the identification of the M-protein from the accurate molecular mass of the light chain component (39). In a study evaluating the role of delayed M protein clearance, Mills et al. utilized miRAMM (40) and showed that while M-protein positivities at different time points were not associated with shorter PFS and OS, the patients who managed to decrease their M-protein intensities (while in sCR) enjoyed longer PFS and OS compared to those whose values did not change. Finally, MS methods based on MALDI-TOF, despite offering several benefits over immunofixation, do not appear to offer high enough sensitivity for the detection of MRD in patients in CR (41, 42) and, for that reason, are less likely to be as useful in MRD assessment from peripheral blood without significant technical improvements (**Figure 2**). One small study (43) examined 40 post-treatment patients with an MRD(-) BM but not always in conventional CR and found 23 double negative, 3 double-positive, 6 positive only with mass spectrometry, and 8 positive only with BM biopsy when evaluating for MRD. Of the 6 MS positive/NGF negative patients, none progressed during the follow-up, highlighting the confounding effect of the prolonged half-life of M protein. Due to this fact, it is suggested that M protein monitoring for MRD purposes should not rely on a single measurement but the trajectory across multiple tests of the disease course. In another study that utilized a MALDI-TOF method optimized for the detection of serum free light chains, conventional CR or MRD (-) patients with detectable light chains by MS had inferior PFS compared to those without detectable light chains (44). As the optimization of mass spectrometry methods is advancing and greater sensitivities are achieved, their role in MRD assessment should be rigorously evaluated in patients in conventional CR, utilizing high (e.g. miRAMM) and low (e.g. MALDI-TOF) sensitivity mass spectrometry assays as well as gold standards for BM MRD assessment (NGS or NGF).

## Imaging

MM BM infiltration, especially in the post-treatment setting, exhibits substantial spatial heterogeneity with significant implications on the efficacy of a blind BM pull (45). In addition, the prolonged survival of MM patients, along with the development of sensitive imaging tools, have established extramedullary disease assessment as an integral component of MM work-up. Liquid biopsies could, in theory, tackle these problems effectively, but currently, whole-body imaging with either PET-CT or (DWI)-MRI should be used along with the BM studies to determine the most accurate response evaluation (10).

The imaging modality of choice for detecting extramedullary lesions is a PET-CT scan that uses ^18^[F]FDG as the metabolic substrate. Several studies have shown the prognostic significance of a negative PET-CT after induction therapy (46–48). PET negativity is defined as the disappearance of every area of increased tracer uptake found at baseline or a preceding PET-CT or decrease to less mediastinal blood pool SUV or decrease to less than that of surrounding normal tissue per the IMWG (10). For instance, Davies et al. (49) evaluated 596 patients to determine the prognostic significance a negative PET-CT scan has at different time points of treatment: day 7 post-induction, post-induction, post-transplant, and at maintenance. Of note, patients with complete resolution of initial PET-CT lesions 7 days after induction therapy had the same outcomes as those with no lesions at baseline. The superior prognostic effect of subsequent disease resolutions was retained at all time points.

Regarding MRD concordance with PET-CT negativity, Rasche et al. (50) showed that 12% of patients found to be MRD negative following induction therapy still had hypermetabolic focal lesions on PET-CT. These patients had a worse PFS than double-negative patients. This discrepancy was increased in the salvage therapy setting, with 50% of MRD(-) patients with flow cytometry still having PET-CT avid lesions, highlighting the heterogeneous behavior of MM after successive drug therapies. Similar discordance was seen in the CASSIOPEIA study (51) and other studies with many patients that are PET-CT negative still being MRD(+) in the BM whereas less than a quarter of patients that are BM MRD(-) demonstrating PET avid lesions (46, 52). These findings are mainly attributed to the heterogeneous biologic behavior of MM, which is especially prevalent in the post-treatment setting. An example of discordant results would be an extramedullary plasmacytoma, which can only be captured by PET-CT, as MRD assessment in the BM would likely be negative. In addition, the spatial infiltration of BM by plasma cells can introduce false-negative results in the biopsy, and imaging-guided biopsy will be the only avenue for true disease assessment. Conversely, because of the minute plasma cell quantities detected by MRD assays (e.g., 1 abnormal cell in 10 million), PET-CT will likely be negative for these patients since detection thresholds are limited in lesions under 5mm. Clinically, due to these methods’ different disease prisms (infinitesimal quantities of plasma cells in the Bone Marrow for MRD and broad, whole-body evaluation for PET-CT), information from both is required to comprehensively evaluate residual malignancy, and case-by-case treatment decisions should be employed. Recent efforts were targeted at standardizing the PET-CT results, and the recently validated Deavillue criteria originally used for lymphoma should be employed in the reports (53).

The use of conventional MRI has been somewhat reduced with the advent of PET-CT in the post-treatment setting, primarily because MRI cannot reliably distinguish active lesions from healthy bone remodeling after chemotherapy (54). More specifically, the major disadvantage for MRI in the post-treatment setting is the delayed capture of treatment response, owing to the lack of differentiation between vital and necrotic tissue in previous osteolytic lesions. This fact was highlighted when Davies et al. compared the utility of PET-CT *vs.* whole-body MRI to determine remission status post-treatment. They showed that although MRI had a higher sensitivity for detecting residual lesions than PET-CT (80% *vs.* 50%), the false-positive rate was also significantly higher for MRI (61.9% *vs.* 14.3%, respectively). As a result, the prognostic role of MRI over PET/CT remains doubtful (55, 56). In the IFM/DFCI 2009 trial, achieving an MRI negative state after induction with 3 cycles of RVD and before maintenance was not prognostic, whereas PET/CT negativity predicted better PFS and OS (30-month PFS, 78.7% *vs* 56.8%, respectively (46). While not adequately standardized, Diffusion-Weighted MRI has emerged as a potential sensitive marker with evidence showing comparable or better sensitivity than PET-CT (50, 57, 58). The same investigators mentioned above (50) reported a higher rate of (DWI)-MRI positivity of focal lesions compared to PET-CT(21% *vs* 6%), but importantly not all PET-CT positive patients were MRI positive too, suggesting the complementary role of these imaging modalities. Discordance may be due to the relatively common hexokinase deficiency, the enzyme that catalyzes glucose phosphorylation upon cell entry, a major reason for false-negative results for the ^18^F molecule, with daratumumab and BCMA(B-cell maturation antigen) based substrates being studied as alternative immunologic options (59–61). Conversely, differences in the bone microenvironment and cellularity, which are associated with increased age and necrosis (caused by ongoing treatment), can influence the imaging pattern and thus limit the use of (DWI)-MRI for metabolic response assessment (62). While further corroboration is warranted to support the regular use of DWI-MRI in MM patients, ^18^[F]FDG PET-CT remains the most practical option for MRD detection and should be monitored at pre-specified time points and in a standardized manner (e.g., the recently validated Deauville score for MM).

## Association Between MRD Negative Status and Prognosis

The prognostic value of achieving undetectable MRD is now supported by several studies in addition to those mentioned so far. Goicoechea et al. showed that patients attaining MRD negativity after therapy have similar, improved outcomes irrespective of disease risk at diagnosis (63). Other studies (11, 64–66) have supported this finding, with initial risk stratification by R-ISS, FISH, ISS retaining prognostic significance only in the subset of high-risk patients who do not reach MRD negativity (18). More specifically, Li et al. (65) showed that while both high-risk disease characteristics and MRD positivity are independent unfavorable factors for myeloma, no significant difference was seen in PFS or OS between the MRD(-) high-risk group and MRD(+) standard-risk group (median PFS 45 *vs.* 34 m, *P* = 0.300; 4-year OS 100% *vs.* 83.6%, *P* = 0.196).

The current body of literature is primarily comprised of studies evaluating newly diagnosed MM patients, with clear associations between MRD(-) and longer PFS and OS (67–70). This is especially true when MRD is reached in the pre-transplant evaluation, compared to 100 days post-transplant, which is likely reflective of more responsive disease biology overall (71–77). Rawstron et al. (71) evaluated MRD after induction therapy and 100 days post-transplant and showed significantly prolonged PFS (median PFS, 44.2 months; *P* <.001) in patients with early MRD negativity, compared with the patients that were MRD(-) after ASCT. However, the patients who received transplants had a 2.8-4.2 fold increase in the probability of achieving an MRD(-) state, underscoring the role of transplant in patients who do not respond as deeply with induction therapy. The same observation was highlighted by Paiva et al. (73), with significant PFS and OS increases seen in the group of patients who respond before consolidation and transplant.

In the largest meta-analysis to date, Munshi et al. (78) examined 44 studies with eligible MRD data for PFS and 23 studies for OS. They showed that achieving MRD negativity led to improved PFS (HR, 0.33; 95% CI, 0.29-0.37; P <.001) and OS (HR, 0.45; 95% CI, 0.39-0.51; P <.001), irrespective of several disease and patient characteristics. The beneficial effect of MRD(-) was sustained, regardless of MRD sensitivity thresholds (older studies reported results with sensitivity of 10^-4^), cytogenetic risk, method of MRD assessment, depth of clinical response at the time of MRD measurement, and MRD assessment premaintenance and 12 months after the start of maintenance therapy. PFS estimates were more favorable if MRD negativity was achieved at 12 months post-maintenance than pre-maintenance, highlighting the intuitive correlation between the depth and duration of response. Although the prognostic effect that the MRD(-) state has on newly diagnosed MM was described by previous trials, one of the most important findings of this meta-analysis was the effect shown on the relapsed, heavily pre-treated population, with MRD negative patients having improved PFS (HR, 0.34; 95% CI, 0.24-0.47; P<.001); and OS (HR, 0.28; 95% CI, 0.18-0.45; P <.001) (79, 80). This finding was reinforced when a pooled analysis of the POLLUX and CASTOR trials was done by Avet-Loiseau et al. and showed that daratumumab-based regimens can lead to MRD(-) remissions in relapsed patients, which translated to improved PFS (81). This is especially true in the subgroup of patients with sustained MRD negativity (6-12, >12 months) since significantly prolonged clinical outcomes were seen in both studies regardless of assigned treatment. However, sustained MRD negativity can be an impractical endpoint, since a) any level of sustained remission will be associated with improved outcomes and b) it will require more time to “read out” as a clinical trial endpoint, thereby eliminating the potential utility of MRD negativity as an early surrogate for more traditional endpoints such as PFS and OS. Higher MRD (-) after induction, consolidation and at 12 months after start of therapy was noted in the experimental arm of the CASSIOPEIA trial comparing bortezomib, thalidomide dexamethasone with or without daratumumab followed by transplant and daratumumab consolidation or maintenance after a second randomization. MRD negativity was associated with improved outcomes irrespective of treatment type (82).

Conversely, in a phase 1 study of 33 patients with relapsed refractory heavily pre-treated MM, bb2121 targeting CAR-T therapy was infused. Of the 16 patients eligible for MRD evaluation (partial response or better), 15 were negative at the first BM biopsy (94%). However, 6 of them relapsed in the 11.3 median follow-up period, suggesting different disease kinetics and response patterns in patients treated with drugs with novel mechanisms of action and with more advanced disease (83).

MM patients display significant treatment response variability, leaving our current approaches one step behind the evasive nature of the disease. In the majority of MM patients, regardless of the stratification risk, a relatively uniform therapeutic regimen is applied (induction, transplant, post-transplant consolidation, and/or maintenance). However, sequential MRD assessments may allow for a better understanding of the efficacy of different therapeutic agents their relevance to the particular disease genotype-phenotype and perhaps allow for escalation or de-escalation of therapy based on MRD status. Such an approach may limit the financial burden and medical toxicity for the subset of patients that could do as well without receiving as intensive a therapy.

The ability to identify patients with the deepest responses will also help optimize the existing diagnostic risk assessment tools for MM patients. Risk stratification is primarily based on initial cytogenetic aberrations in the malignant clone population, with specific mutations, such as del17p, portraying the worst outcomes. However, significant unpredictability still exists, with some high-risk patients following less aggressive disease courses and vice versa. Therefore, it is essential to properly evaluate the disease characteristics of these patients, and sustainable MRD negativity in the response assessment should add additional insight as to which factors influence the prognostic algorithm in MM.

In an attempt to provide international consensus guidance on assessing and reporting MRD in MM clinical trials, a panel of experts in the MM field published recommendations aiming to harmonize the published data and improve MRD research quality worldwide (84). The panelists recognized that while consensus statements could be provided on timing and methodology, multiple clinical trials are vital if incorporation of MRD assessment is to be contemplated as a surrogate for clinical decisions in the care of MM patients. Among the consensus statements they suggested, MRD evaluation must be analytically validated on the level of assay’s technical performance (as far as its limit of blank (LOB), limit of detection (LOD), and limit of quantification (LOQ) is concerned, the sequence and quality of BM sample) and clearly reported in clinical trials. Regarding peripheral blood MRD assays, the need for further investigation and cross-validation versus BM-based MRD is emphasized. Considering the additional prognostic information obtained by the various imaging techniques, the authors recommend PET-CT and consider it complementary to BM MRD assessment. The panelists also advocate MRD assessment in all trials for newly diagnosed MM patients as well as smoldering MM with curative intent and trials for relapsed or refractory MM. Regarding the timing of assessment, they proposed that for the NDMM patient clinical trials, MRD be assessed at the end of induction, post-AHCT (when relevant), before maintenance, and periodically thereafter. While for clinical trials without distinct therapeutic phases, MRD is to be performed whenever a BM biopsy is attempted to assess treatment response.

## Barriers in Incorporating MRD Testing in Clinical Practice

Several practical barriers remain prior to wide applicability of MRD in clinical practice. The association between MRD and PFS and OS in newly diagnosed patients and MRD and PFS in relapsed disease is clear. This is not surprising. For MRD to become a robust surrogate for drug approval in this setting, specific thresholds for changes in MRD(-) rates need to be defined. In other words, “how much more MRD negativity” is necessary for a drug to *consistently* lead to improved PFS and, ideally, OS so that MRD(-) can be used as a surrogate for these clinically relevant endpoints. And also, in what setting? Since MRD “benefit-cutoffs” will likely be different for newly diagnosed versus relapsed disease or for high risk versus non-high risk disease (likely “more” MRD negativity will be required in the latter scenarios).

Furthermore, the myeloma community needs to stay consistent with an optimal threshold of sensitivity, which is more important than the actual method used to reach that threshold. MRD will need to be tested in all patients after achieving conventional CR but not patients achieving fewer deep responses. Current studies are sometimes limited by the use of methods with various sensitivity thresholds, used in patients in less than CR, and with missing MRD assessments in some cases, which limits the generalizability of the results. In the case of missing MRD status, IMWG has suggested that these patients be considered MRD(+) as a more conservative approach. The timing of MRD assessment needs to be considered since the issue mentioned above may differ according to various time points (e.g., newly diagnosed versus relapsed disease). This is also relevant for patients according to cytogenetic risk status, which should also be considered since patients with high-risk disease may achieve MRD(-) complete remissions that are relatively short-lived compared to their standard risk counterparts. To support this claim, Diamond et al. showed that high-risk patients who achieved and subsequently lost MRD negativity had a worse PFS compared to patients with unremitting, low MRD positive rates throughout the treatment follow-up. These findings underscore the importance of sustained biochemical and imaging responses, especially in the high-risk populations since early relapse from MRD(-) is consistently associated with inferior outcomes (85). Further, in the relapsed setting, the phase 3 BELLINI trial showed that while the addition of Venetoclax to Bortezomib and dexamethasone significantly improved median PFS and MRD negativity rates, it was also associated with increased overall mortality *vs.* Bortezomib and dexamethasone (86). This was likely secondary to higher rates of infections in the treatment arm and highlights the limitations of surrogate endpoints in general.

Finally, the implications of using MRD with several of the new immunotherapeutics (such as CART cells) are not clear and may need to be validated separately considering the significantly different mechanism of action of these approaches compared to conventional MM therapies.

When it comes to imaging studies, the exact incremental prognostic value over BM-based approaches is not entirely clarified, as is the optimal timing of obtaining imaging after therapy or with relapsed disease. Ideally, accurate MRD assessment should include both a sensitive BM-focused approach and PET/CT imaging in all patients.

The use of MRD to inform treatment decisions will require rigorous testing in a clinical trial setting. We do not know yet in which setting we should escalate therapy to help patients reach an MRD- status or if we could de-escalate therapy in patients achieving sustained MRD. Both approaches are actively studied in ongoing studies (**Table 3**). For instance, while the benefit of ASCT has been extensively validated for newly diagnosed, fit MM patients (87, 88) in terms of PFS, the IFM 2009 study showed no difference in PFS for MRD(-) patients irrespective of the treatment following induction (ASCT or VRD) (11). It should be noted that more patients in the transplant receiving group achieved MRD negativity (30% *vs.* 21%). In 2019, Gay et al. showed no difference in the MRD negativity (defined as 10^-5^ by NGS) rate between the group that received induction with carfilzomib-lenalidomide-dexamethasone (KRD) followed by ASCT and consolidation with KRD and 12 cycles of KRD without ASCT (58% *VS* 54% respectively) (89). This is also shown in other studies that suggested that upfront 4 drug regimens(e.g., daratumumab-KRD) without subsequent transplantation can achieve comparable MRD results with patients that receive transplant following induction (90, 91). Although additional validation is essential in order to safely use MRD as a marker that will guide clinical decisions, the above evidence indicates that more intensive treatment approaches may be withheld in patients with highly responsive disease biology.

**Table 3:** Selected trials with MRD status guiding intensification or de-intensification of therapy.

Title	Intervention/description	Phase	Estimated Primary completion date	Primary endpoint	Brief outline
NCT04071457 DRAMMATIC study	Drug: Lenalidomide Drug: Daratumumab/rHuPH20	Phase 3	July 1, 2029	Overall Survival	After 2 years of maintenance, patients are assessed by MRD, and positive patients will continue with the assigned treatment. MRD negative patients will be further randomized to continue/discontinue treatment
NCT04513639 REMNANT study	Drug: Early treatment of relapse with carfilzomib, dexamethasone, daratumumab Drug: Standard treatment of relapse with carfilzomib, dexamethasone, daratumumab	Phase 2- 3	June 1, 2030	PFS, OS, MRD negativity after first-line treatment	Newly diagnosed patients will be treated with standard induction. Patients that reach MRD negativity will be randomized and evaluate whether treating minimal residual disease (MRD) relapse after first-line treatment prolongs progression-free survival and overall survival for myeloma patients versus treating relapse after progressive disease
NCT04140162	MRD Driven Adaptive Strategy in Treatment for Newly Diagnosed MM With Upfront Daratumumab-based Therapy	Phase 2	October 1, 2024	Proportion of participants who reach MRD negativity upfront or after consolidation	This phase 2 trial will test whether the combination of DaraRd (daratumumab + lenalidomide + dexamethasone) as induction therapy, followed by DRVd (daratumumab + lenalidomide + bortezomib + dexamethasone) consolidation therapy, if needed, will result in more patients achieving minimal residual disease MRD negative status, relative to the standard of care. Consolidation therapy will be administered only to those patients with MRD positive status after induction therapy.
NCT03992170, DART4MM study	Efficacy of Daratumumab in MM Patients in >VGPR/MRD positive by Next Generation Flow	Phase 2	June 30, 2022	Overall Response Rate (ORR)	MRD positive but >VGPR patients at 6 months of therapy will continue Daratumumab for 2 years. MRD negative patients can stop treatment
NCT03901963 AURIGA study	A Randomized Study of Daratumumab Plus Lenalidomide Versus Lenalidomide Alone as Maintenance Treatment in Patients With Newly Diagnosed MM who Are Minimal Residual Disease Positive After Frontline Autologous Stem Cell Transplant	Phase 3	October 31, 2023	MRD negativity	Evaluation of the conversion rate of MRD positive patients to MRD negative in the maintenance phase between Daratumumab-Lenalidomide and Lenalidomide alone
NCT03224507 MASTER trial	Drug: KRdD followed by auto-HCT Drug: KRdD only	Phase 2	April 1, 2022	MRD negativity at the completion of consolidation	After induction therapy with KRdD (Kyprolis, Revlimid, dexamethasone, Darzalex), duration of consolidation and maintenance therapy will be guided by MRD rates. Patients with MRD (-) at or after cycle 1 of consolidation will be actively monitored.

**Table 4** summarizes the current limitations of MRD in clinical decision-making and highlights some potential issues that should be targeted by ongoing and future trials.

**Table 4:** Barriers for regular use and future prospects of MRD assessment in MM.

1. What is the most appropriate sensitivity threshold (10^-5^,10^-6^, or higher) to determine MRD presence? Is an exact quantifying report more prognostically useful than a cutoff of positivity/negativity?
2. Does the speed of negative MRD attainment matter clinically? Are high-risk patients more prone to lose the MRD negativity compared to standard-risk patients? Should MRD- cutoffs be different according to disease biology?
3. Should clinicians intensify or de-intensify their therapeutic approaches based on MRD results at different time points? If yes, what is the optimal point of disease assessment that MRD negativity should be pursued more vigorously?
4. Can blood-based methods replace or complement the laborious MRD assessment in the marrow? Should patients with extramedullary disease be followed with imaging, bone marrow, and blood-based studies? How often should patients with sustained MRD negativity (over a year) be followed?
5. Can MRD guide decisions as to whether prolonged maintenance therapies should be given indefinitely? Is the loss of MRD negativity a justified event of re-starting treatment without overt clinical, biochemical signs?
6. Is MRD a clinically relevant target that can justify the use of highly toxic therapies (CAR-T) instead of the traditional markers (PFS, OS)?
7. Are there tumor-extrinsic factors that can explain durable MRD negative responses (immunome, microbiome, etc.)?

Finally, MRD(-) patients by BM testing and PET/CT should be studied further. Why do these patients relapse? Is it really a pool of plasma cells that remain undetectable at such low levels that give rise to relapsed disease? Or is the source of relapse phenotypically or perhaps genomically very different than what we typically consider a malignant plasma cell? In other words, are we looking for the right cell in this setting? The study of these patients and the answer to this question may shed more light on the path to MM cure.

## Author Contributions

Both authors conceived the manuscript content, edited, and critically revised the manuscript.

## Conflict of Interest

The authors declare that the research was conducted in the absence of any commercial or financial relationships that could be construed as a potential conflict of interest.

## Publisher’s Note

All claims expressed in this article are solely those of the authors and do not necessarily represent those of their affiliated organizations, or those of the publisher, the editors and the reviewers. Any product that may be evaluated in this article, or claim that may be made by its manufacturer, is not guaranteed or endorsed by the publisher.

## References

[1] Facon T Kumar S Plesner T Orlowski RZ Moreau P Bahlis N. Daratumumab Plus Lenalidomide and Dexamethasone for Untreated Myeloma. N Engl J Med (2019) 380(22):2104–15. doi: 10.1056/NEJMoa1817249 PMC10045721 31141632

[2] Voorhees PM Kaufman JL Laubach J Sborov DW Reeves B Rodriguez C. Daratumumab, Lenalidomide, Bortezomib, and Dexamethasone for Transplant-Eligible Newly Diagnosed Multiple Myeloma: The GRIFFIN Trial. Blood (2020) 136(8):936–45. doi: 10.1182/blood.2020005288 PMC7441167 32325490

[3] Kapoor P Kumar SK Dispenzieri A Lacy MQ Buadi F Dingli D. Importance of Achieving Stringent Complete Response After Autologous Stem-Cell Transplantation in Multiple Myeloma. J Clin Oncol (2013) 31(36):4529–35. doi: 10.1200/JCO.2013.49.0086 PMC3871514 24248686

[4] Sidana S Tandon N Dispenzieri A Gertz MA Buadi FK Lacy MQ. Relapse After Complete Response in Newly Diagnosed Multiple Myeloma: Implications of Duration of Response and Patterns of Relapse. Leukemia (2019) 33(3):730–8. doi: 10.1038/s41375-018-0271-1 30323358

[5] van de Velde HJ Liu X Chen G Cakana A Deraedt W Bayssas M. Complete Response Correlates With Long-Term Survival and Progression-Free Survival in High-Dose Therapy in Multiple Myeloma. Haematologica (2007) 92(10):1399–406. doi: 10.3324/haematol.11534 18024376

[6] Korde N Roschewski M Zingone A Kwok M Manasanch EE Bhutani M. Treatment With Carfilzomib-Lenalidomide-Dexamethasone With Lenalidomide Extension in Patients With Smoldering or Newly Diagnosed Multiple Myeloma. JAMA Oncol (2015) 1(6):746–54. doi: 10.1001/jamaoncol.2015.2010 PMC6662597 26181891

[7] Jakubowiak AJ Dytfeld D Griffith KA Lebovic D Vesole DH Jagannath S. A Phase 1/2 Study of Carfilzomib in Combination With Lenalidomide and Low-Dose Dexamethasone as a Frontline Treatment for Multiple Myeloma. Blood (2012) 120(9):1801–9. doi: 10.1182/blood-2012-04-422683 PMC5162553 22665938

[8] Mateos MV Cavo M Blade J Dimopoulos MA Suzuki K Jakubowiak A. Overall Survival With Daratumumab, Bortezomib, Melphalan, and Prednisone in Newly Diagnosed Multiple Myeloma (ALCYONE): A Randomised, Open-Label, Phase 3 Trial. Lancet (2020) 395(10218):132–41. doi: 10.1016/S0140-6736(19)32956-3 31836199

[9] Avet-Loiseau H Lauwers-Cances V Corre J Moreau P Attal M Munshi NC. Minimal Residual Disease in Multiple Myeloma: Final Analysis of the IFM2009 Trial. Blood (2017) 130:435–5. doi: 10.1182/blood.V130.Suppl_1.435.435

[10] Kumar S Paiva B Anderson KC Durie B Landgren O Moreau P. International Myeloma Working Group Consensus Criteria for Response and Minimal Residual Disease Assessment in Multiple Myeloma. Lancet Oncol (2016) 17(8):e328–46. doi: 10.1016/s1470-2045(16)30206-6 27511158

[11] Perrot A Lauwers-Cances V Corre J Robillard N Hulin C Chretien ML. Minimal Residual Disease Negativity Using Deep Sequencing is a Major Prognostic Factor in Multiple Myeloma. Blood (2018) 132(23):2456–64. doi: 10.1182/blood-2018-06-858613 PMC6284215 30249784

[12] Solovev MV Mendeleeva LP Firsova MV Galtseva IV Davydova J Gemdzhian EG. Efficacy of Maintenance Therapy Following Auto-HSCT Depending on MRD Status in Patients With Multiple Myeloma. Blood (2018) 132(Supplement 1):3432–2. doi: 10.1182/blood-2018-99-112790

[13] Mateos MV Oriol A Martínez-López J Teruel AI López de la Guía A López J. GEM2005 Trial Update Comparing VMP/VTP as Induction in Elderly Multiple Myeloma Patients: Do We Still Need Alkylators? Blood (2014) 124(12):1887–93. doi: 10.1182/blood-2014-05-573733 25102853

[14] van Dongen JJ Lhermitte L Böttcher S Almeida J van der Velden VH Flores-Montero J. EuroFlow Antibody Panels for Standardized N-Dimensional Flow Cytometric Immunophenotyping of Normal, Reactive and Malignant Leukocytes. Leukemia (2012) 26(9):1908–75. doi: 10.1038/leu.2012.120 PMC3437410 22552007

[15] Flores-Montero J Sanoja-Flores L Paiva B Puig N García-Sánchez O Böttcher S. Next Generation Flow for Highly Sensitive and Standardized Detection of Minimal Residual Disease in Multiple Myeloma. Leukemia (2017) 31(10):2094–103. doi: 10.1038/leu.2017.29 PMC5629369 28104919

[16] Avet-Loiseau H Corre J Lauwers-Cances V Chretien M-L Robillard N Leleu X. Evaluation of Minimal Residual Disease (MRD) By Next Generation Sequencing (NGS) Is Highly Predictive of Progression Free Survival in the IFM/DFCI 2009 Trial. Blood (2015) 126:191. doi: 10.1182/blood.V126.23.191.191

[17] Roshal M Flores-Montero JA Gao Q Koeber M Wardrope J Durie BGM. MRD Detection in Multiple Myeloma: Comparison Between MSKCC 10-Color Single-Tube and EuroFlow 8-Color 2-Tube Methods. Blood Adv (2017) 1(12):728–32. doi: 10.1182/bloodadvances.2016003715 PMC5728052 29296716

[18] Paiva B Puig N Cedena MT Rosiñol L Cordón L Vidriales MB. Measurable Residual Disease by Next-Generation Flow Cytometry in Multiple Myeloma. J Clin Oncol (2020) 38(8):784–92. doi: 10.1200/jco.19.01231 31770060

[19] Rustad EH Misund K Bernard E Coward E Yellapantula VD Hultcrantz M. Stability and Uniqueness of Clonal Immunoglobulin CDR3 Sequences for MRD Tracking in Multiple Myeloma. Am J Hematol (2019) 94(12):1364–73. doi: 10.1002/ajh.25641 PMC7449571 31571261

[20] Ho C Arcila ME. Minimal Residual Disease Detection of Myeloma Using Sequencing of Immunoglobulin Heavy Chain Gene VDJ Regions. Semin Hematol (2018) 55(1):13–8. doi: 10.1053/j.seminhematol.2018.02.007 29759147

[21] Ching T Duncan ME Newman-Eerkes T McWhorter MME Tracy JM Steen MS. Analytical Evaluation of the clonoSEQ Assay for Establishing Measurable (Minimal) Residual Disease in Acute Lymphoblastic Leukemia, Chronic Lymphocytic Leukemia, and Multiple Myeloma. BMC Cancer (2020) 20(1):612. doi: 10.1186/s12885-020-07077-9 32605647 PMC7325652

[22] Bakkus MH Bouko Y Samson D Apperley JF Thielemans K Van Camp B. Post-Transplantation Tumour Load in Bone Marrow, as Assessed by Quantitative ASO-PCR, Is a Prognostic Parameter in Multiple Myeloma. Br J Haematol (2004) 126(5):665–74. doi: 10.1111/j.1365-2141.2004.05120.x 15327517

[23] Ladetto M Brüggemann M Monitillo L Ferrero S Pepin F Drandi D. Next-Generation Sequencing and Real-Time Quantitative PCR for Minimal Residual Disease Detection in B-Cell Disorders. Leukemia (2014) 28(6):1299–307. doi: 10.1038/leu.2013.375 24342950

[24] Puig N Sarasquete ME Balanzategui A Martínez J Paiva B García H. Critical Evaluation of ASO RQ-PCR for Minimal Residual Disease Evaluation in Multiple Myeloma. A Comparative Analysis With Flow Cytometry. Leukemia (2014) 28(2):391–7. doi: 10.1038/leu.2013.217 23860448

[25] Takamatsu H. Comparison of Minimal Residual Disease Detection by Multiparameter Flow Cytometry, ASO-qPCR, Droplet Digital PCR, and Deep Sequencing in Patients With Multiple Myeloma Who Underwent Autologous Stem Cell Transplantation. J Clin Med (2017) 6(10):91. doi: 10.3390/jcm6100091 PMC5664006 28946710

[26] Oliva S Genuardi E Belotti A Frascione PMM Galli M Capra A. Minimal Residual Disease Evaluation By Multiparameter Flow Cytometry and Next Generation Sequencing in the Forte Trial for Newly Diagnosed Multiple Myeloma Patients. Blood (2019) 134(Supplement_1):4322–2. doi: 10.1182/blood-2019-124645

[27] Martinez-Lopez J Lahuerta JJ Pepin F González M Barrio S Ayala R. Prognostic Value of Deep Sequencing Method for Minimal Residual Disease Detection in Multiple Myeloma. Blood (2014) 123(20):3073–9. doi: 10.1182/blood-2014-01-550020 PMC4023416 24646471

[28] Medina A Puig N Flores-Montero J Jimenez C Sarasquete ME Garcia-Alvarez M. Comparison of Next-Generation Sequencing (NGS) and Next-Generation Flow (NGF) for Minimal Residual Disease (MRD) Assessment in Multiple Myeloma. Blood Cancer J (2020) 10(10):108. doi: 10.1038/s41408-020-00377-0 33127891 PMC7603393

[29] Mithraprabhu S Chen M Savvidou I Reale A Spencer A. Liquid Biopsy: An Evolving Paradigm for the Biological Characterisation of Plasma Cell Disorders. Leukemia (2021) 35(10):2771–83. doi: 10.1038/s41375-021-01339-6 34262132

[30] Sanoja-Flores L Flores-Montero J Puig N Contreras-Sanfeliciano T Pontes R Corral-Mateos A. Blood Monitoring of Circulating Tumor Plasma Cells by Next Generation Flow in Multiple Myeloma After Therapy. Blood (2019) 134(24):2218–22. doi: 10.1182/blood.2019002610 PMC6966491 31697808

[31] Oberle A Brandt A Voigtlaender M Thiele B Radloff J Schulenkorf A. Monitoring Multiple Myeloma by Next-Generation Sequencing of V(D)J Rearrangements From Circulating Myeloma Cells and Cell-Free Myeloma DNA. Haematologica (2017) 102(6):1105–11. doi: 10.3324/haematol.2016.161414 PMC5451343 28183851

[32] Gonsalves WI Rajkumar SV Gupta V Morice WG Timm MM Singh PP. Quantification of Clonal Circulating Plasma Cells in Newly Diagnosed Multiple Myeloma: Implications for Redefining High-Risk Myeloma. Leukemia (2014) 28(10):2060–5. doi: 10.1038/leu.2014.98 PMC4162866 24618735

[33] Li J Wang N Tesfaluul N Gao X Liu S Yue B. Prognostic Value of Circulating Plasma Cells in Patients With Multiple Myeloma: A Meta-Analysis. PloS One (2017) 12(7):e0181447. doi: 10.1371/journal.pone.0181447 28704521 PMC5509371

[34] Rajshekhar C Eli M Shaji KK Dragan J Francis KB David D. Serial Measurements of Circulating Plasma Cells Before and After Induction Therapy Have an Independent Prognostic Impact in Patients With Multiple Myeloma Undergoing Upfront Autologous Transplantation. Haematologica (2017) 102(8):1439–45. doi: 10.3324/haematol.2017.166629 PMC5541877 28473618

[35] Kumar S Witzig TE Greipp PR Rajkumar SV. Bone Marrow Angiogenesis and Circulating Plasma Cells in Multiple Myeloma. Br J Haematol (2003) 122(2):272–4. doi: 10.1046/j.1365-2141.2003.04428.x 12846897

[36] Paiva B Paino T Sayagues JM Garayoa M San-Segundo L Martín M. Detailed Characterization of Multiple Myeloma Circulating Tumor Cells Shows Unique Phenotypic, Cytogenetic, Functional, and Circadian Distribution Profile. Blood (2013) 122(22):3591–8. doi: 10.1182/blood-2013-06-510453 24072855

[37] Mazzotti C Buisson L Maheo S Perrot A Chretien ML Leleu X. Myeloma MRD by Deep Sequencing From Circulating Tumor DNA Does Not Correlate With Results Obtained in the Bone Marrow. Blood Adv (2018) 2(21):2811–3. doi: 10.1182/bloodadvances.2018025197 PMC6234381 30355580

[38] Tapia-Alveal C Olsen TR Worgall TS. Personalized Immunoglobulin Aptamers for Detection of Multiple Myeloma Minimal Residual Disease in Serum. Commun Biol (2020) 3(1):781. doi: 10.1038/s42003-020-01515-x 33335255 PMC7747622

[39] Barnidge DR Dasari S Botz CM Murray DH Snyder MR Katzmann JA. Using Mass Spectrometry to Monitor Monoclonal Immunoglobulins in Patients With a Monoclonal Gammopathy. J Proteome Res (2014) 13(3):1419–27. doi: 10.1021/pr400985k 24467232

[40] Mills JR Barnidge DR Dispenzieri A Murray DL. High Sensitivity Blood-Based M-Protein Detection in sCR Patients With Multiple Myeloma. Blood Cancer J (2017) 7(8):e590. doi: 10.1038/bcj.2017.75 28841203 PMC5596386

[41] Foureau D Bhutani M Guo F Rigby K Leonidas M Tjaden E. Comparison of Mass Spectrometry and Flow Cytometry in Measuring Minimal Residual Disease in Multiple Myeloma. Cancer Med (2021) 10(20):6933–6. doi: 10.1002/cam4.4254 PMC8525140 34494717

[42] Milani P Murray DL Barnidge DR Kohlhagen MC Mills JR Merlini G. The Utility of MASS-FIX to Detect and Monitor Monoclonal Proteins in the Clinic. Am J Hematol (2017) 92(8):772–9. doi: 10.1002/ajh.24772 28439985

[43] Eveillard M Rustad E Roshal M Zhang Y Ciardiello A Korde N. Comparison of MALDI-TOF Mass Spectrometry Analysis of Peripheral Blood and Bone Marrow-Based Flow Cytometry for Tracking Measurable Residual Disease in Patients With Multiple Myeloma. Br J Haematol (2020) 189(5):904–7. doi: 10.1111/bjh.16443 PMC7275888 32026474

[44] Giles HV Drayson MT Wright N Cook G Davies FE Morgan GJ. 820 Residual Monoclonal Free Light Chain Positivity By Mass Spectrometry Identifies Patients at Increased Risk of Early Relapse Following First-Line Anti-Myeloma Treatment. Atlanta, GA, United States: ASH (2021).

[45] Rasche L Kortüm KM Raab MS Weinhold N. The Impact of Tumor Heterogeneity on Diagnostics and Novel Therapeutic Strategies in Multiple Myeloma. Int J Mol Sci (2019) 20(5):1248. doi: 10.3390/ijms20051248 PMC6429294 30871078

[46] Moreau P Attal M Caillot D Macro M Karlin L Garderet L. Prospective Evaluation of Magnetic Resonance Imaging and [(18)F]Fluorodeoxyglucose Positron Emission Tomography-Computed Tomography at Diagnosis and Before Maintenance Therapy in Symptomatic Patients With Multiple Myeloma Included in the IFM/DFCI 2009 Trial: Results of the IMAJEM Study. J Clin Oncol (2017) 35(25):2911–8. doi: 10.1200/JCO.2017.72.2975 PMC5578392 28686535

[47] Bartel TB Haessler J Brown TL Shaughnessy JD Jr van Rhee F Anaissie E. F18-Fluorodeoxyglucose Positron Emission Tomography in the Context of Other Imaging Techniques and Prognostic Factors in Multiple Myeloma. Blood (2009) 114(10):2068–76. doi: 10.1182/blood-2009-03-213280 PMC2744568 19443657

[48] Nanni C Zamagni E Celli M Caroli P Ambrosini V Tacchetti P. The Value of 18F-FDG PET/CT After Autologous Stem Cell Transplantation (ASCT) in Patients Affected by Multiple Myeloma (MM): Experience With 77 Patients. Clin Nucl Med (2013) 38(2):e74–9. doi: 10.1097/RLU.0b013e318266cee2 23143049

[49] Davies FE Rosenthal A Rasche L Petty NM McDonald JE Ntambi JA. Treatment to Suppression of Focal Lesions on Positron Emission Tomography-Computed Tomography Is a Therapeutic Goal in Newly Diagnosed Multiple Myeloma. Haematologica (2018) 103(6):1047–53. doi: 10.3324/haematol.2017.177139 PMC6058800 29567784

[50] Rasche L Alapat D Kumar M Gershner G McDonald J Wardell CP. Combination of Flow Cytometry and Functional Imaging for Monitoring of Residual Disease in Myeloma. Leukemia (2019) 33(7):1713–22. doi: 10.1038/s41375-018-0329-0 PMC6586541 30573775

[51] Moreau P Zweegman S Perrot A Hulin C Caillot D Facon T. Evaluation of the Prognostic Value of Positron Emission Tomography-Computed Tomography (PET-CT) at Diagnosis and Follow-Up in Transplant-Eligible Newly Diagnosed Multiple Myeloma (TE NDMM) Patients Treated in the Phase 3 Cassiopeia Study: Results of the Cassiopet Companion Study. Blood (2019) 134(Supplement_1):692–2. doi: 10.1182/blood-2019-123143

[52] Alonso R Cedena MT Gómez-Grande A Ríos R Moraleda JM Cabañas V. Imaging and Bone Marrow Assessments Improve Minimal Residual Disease Prediction in Multiple Myeloma. Am J Hematol (2019) 94(8):853–61. doi: 10.1002/ajh.25507 31074033

[53] Zamagni E Nanni C Dozza L Carlier T Bailly C Tacchetti P. Standardization of 18F-FDG–PET/CT According to Deauville Criteria for Metabolic Complete Response Definition in Newly Diagnosed Multiple Myeloma. J Clin Oncol (2021) 39(2):116–25. doi: 10.1200/JCO.20.00386 33151787

[54] Basha MAA Hamed MAG Refaat R AlAzzazy MZ Bessar MA Mohamed EM. Diagnostic Performance of (18)F-FDG PET/CT and Whole-Body MRI Before and Early After Treatment of Multiple Myeloma: A Prospective Comparative Study. Jpn J Radiol (2018) 36(6):382–93. doi: 10.1007/s11604-018-0738-z 29671193

[55] Derlin T Peldschus K Münster S Bannas P Herrmann J Stübig T. Comparative Diagnostic Performance of ¹⁸F-FDG PET/CT Versus Whole-Body MRI for Determination of Remission Status in Multiple Myeloma After Stem Cell Transplantation. Eur Radiol (2013) 23(2):570–8. doi: 10.1007/s00330-012-2600-5 22843058

[56] Spinnato P Bazzocchi A Brioli A Nanni C Zamagni E Albisinni U. Contrast Enhanced MRI and ¹⁸F-FDG PET-CT in the Assessment of Multiple Myeloma: A Comparison of Results in Different Phases of the Disease. Eur J Radiol (2012) 81(12):4013–8. doi: 10.1016/j.ejrad.2012.06.028 22921683

[57] Chen J Li C Tian Y Xiao Q Deng M Hu H. Comparison of Whole-Body DWI and (18)F-FDG PET/CT for Detecting Intramedullary and Extramedullary Lesions in Multiple Myeloma. AJR Am J Roentgenol (2019) 213(3):514–23. doi: 10.2214/AJR.18.20989 31166755

[58] Mesguich C Hulin C Latrabe V Lascaux A Bordenave L Hindié E. Prospective Comparison of 18-FDG PET/CT and Whole-Body Diffusion-Weighted MRI in the Assessment of Multiple Myeloma. Ann Hematol (2020) 99(12):2869–80. doi: 10.1007/s00277-020-04265-2 32951093

[59] Rasche L Angtuaco E McDonald JE Buros A Stein C Pawlyn C. Low Expression of Hexokinase-2 Is Associated With False-Negative FDG–positron Emission Tomography in Multiple Myeloma. Blood (2017) 130(1):30–4. doi: 10.1182/blood-2017-03-774422 PMC5501152 28432222

[60] Kircher S Stolzenburg A Kortüm KM Kircher M Da Via M Samnick S. Hexokinase-2 Expression in (11)C-Methionine-Positive, (18)F-FDG-Negative Multiple Myeloma. J Nucl Med (2019) 60(3):348–52. doi: 10.2967/jnumed.118.217539 30389821

[61] Pawlyn C Fowkes L Otero S Jones JR Boyd KD Davies FE. Whole-Body Diffusion-Weighted MRI: A New Gold Standard for Assessing Disease Burden in Patients With Multiple Myeloma? Leukemia (2016) 30(6):1446–8. doi: 10.1038/leu.2015.338 PMC4895156 26648535

[62] Zamagni E Tacchetti P Barbato S Cavo M. Role of Imaging in the Evaluation of Minimal Residual Disease in Multiple Myeloma Patients. J Clin Med (2020) 9(11):3519. doi: 10.3390/jcm9113519 PMC7692446 33142671

[63] Goicoechea I Puig N Cedena M-T Burgos L Cordón L Vidriales M-B. Deep MRD Profiling Defines Outcome and Unveils Different Modes of Treatment Resistance in Standard- and High-Risk Myeloma. Blood (2021) 137(1):49–60. doi: 10.1182/blood.2020006731 32693406

[64] Lahuerta J-J Paiva B Vidriales M-B Cordón L Cedena M-T Puig N. Depth of Response in Multiple Myeloma: A Pooled Analysis of Three PETHEMA/GEM Clinical Trials. J Clin Oncol (2017) 35(25):2900–10. doi: 10.1200/JCO.2016.69.2517 PMC5568033 28498784

[65] Li H Li F Zhou X Mei J Song P An Z. Achieving Minimal Residual Disease-Negative by Multiparameter Flow Cytometry may Ameliorate a Poor Prognosis in MM Patients With High-Risk Cytogenetics: A Retrospective Single-Center Analysis. Ann Hematol (2019) 98(5):1185–95. doi: 10.1007/s00277-019-03609-x 30721336

[66] Chakraborty R Muchtar E Kumar SK Jevremovic D Buadi FK Dingli D. Impact of Post-Transplant Response and Minimal Residual Disease on Survival in Myeloma With High-Risk Cytogenetics. Biol Blood Marrow Transplant (2017) 23(4):598–605. doi: 10.1016/j.bbmt.2017.01.076 28115277

[67] Gambella M Omedé P Spada S Muccio VE Gilestro M Saraci E. Minimal Residual Disease by Flow Cytometry and Allelic-Specific Oligonucleotide Real-Time Quantitative Polymerase Chain Reaction in Patients With Myeloma Receiving Lenalidomide Maintenance: A Pooled Analysis. Cancer (2019) 125(5):750–60. doi: 10.1002/cncr.31854 30561775

[68] Schinke C Hoering A Wang H Carlton V Thanandrarajan S Deshpande S. The Prognostic Value of the Depth of Response in Multiple Myeloma Depends on the Time of Assessment, Risk Status and Molecular Subtype. Haematologica (2017) 102(8):e313–6. doi: 10.3324/haematol.2017.165217 PMC5541885 28522572

[69] Popat R De Tute RM Counsell N De-Silva D Phillips B Cavenagh JD. Outcomes of Stratification to ASCT or Not Based on Depth of Response: Results of a Phase 2 Trial Assessing the Impact of Minimal Residual Disease (MRD) in Multiple Myeloma Patients With Deferred ASCT (PADIMAC). Blood (2017) 130(Supplement 1):1864–4. doi: 10.1182/blood.V130.Suppl_1.1864.1864

[70] Fukumoto K Fujisawa M Suehara Y Narita KT Usui Y Takeuchi M. Prognostic Impact of Immunophenotypic Complete Response in Patients With Multiple Myeloma Achieving Better Than Complete Response. Leuk Lymphoma (2016) 57(8):1786–92. doi: 10.3109/10428194.2015.1121262 26764045

[71] Rawstron AC Child JA de Tute RM Davies FE Gregory WM Bell SE. Minimal Residual Disease Assessed by Multiparameter Flow Cytometry in Multiple Myeloma: Impact on Outcome in the Medical Research Council Myeloma IX Study. J Clin Oncol (2013) 31(20):2540–7. doi: 10.1200/JCO.2012.46.2119 23733781

[72] Holstein SA Avet-Loiseau H Hahn T Ho CM Lohr JG Munshi NC. BMT CTN Myeloma Intergroup Workshop on Minimal Residual Disease and Immune Profiling: Summary and Recommendations From the Organizing Committee. Biol Blood Marrow Transplant (2018) 24(4):641–8. doi: 10.1016/j.bbmt.2017.12.774 PMC5902638 29242112

[73] Paiva B Vidriales MB Cerveró J Mateo G Pérez JJ Montalbán MA. Multiparameter Flow Cytometric Remission is the Most Relevant Prognostic Factor for Multiple Myeloma Patients Who Undergo Autologous Stem Cell Transplantation. Blood (2008) 112(10):4017–23. doi: 10.1182/blood-2008-05-159624 PMC2581991 18669875

[74] Gu J Liu J Chen M Huang B Li J. Longitudinal Flow Cytometry Identified "Minimal Residual Disease" (MRD) Evolution Patterns for Predicting the Prognosis of Patients With Transplant-Eligible Multiple Myeloma. Biol Blood Marrow Transplant (2018) 24(12):2568–74. doi: 10.1016/j.bbmt.2018.07.040 30142420

[75] Rossi G Falcone AP Minervini MM De Cillis GP De Waure C Sisti LG. Minimal Residual Disease and Log-Reduction of Plasma Cells Are Associated With Superior Response After Double Autologous Stem Cell Transplant in Younger Patients With Multiple Myeloma. Cytometry B Clin Cytom (2019) 96(3):195–200. doi: 10.1002/cyto.b.21755 30549231

[76] Martinez-Lopez J Sanchez-Vega B Barrio S Cuenca I Ruiz-Heredia Y Alonso R. Analytical and Clinical Validation of a Novel in-House Deep-Sequencing Method for Minimal Residual Disease Monitoring in a Phase II Trial for Multiple Myeloma. Leukemia (2017) 31(6):1446–9. doi: 10.1038/leu.2017.58 PMC5467041 28210002

[77] Mateos MV Dimopoulos MA Cavo M Suzuki K Jakubowiak A Knop S. Daratumumab Plus Bortezomib, Melphalan, and Prednisone for Untreated Myeloma. N Engl J Med (2018) 378(6):518–28. doi: 10.1056/NEJMoa1714678 29231133

[78] Munshi NC Avet-Loiseau H Anderson KC Neri P Paiva B Samur M. A Large Meta-Analysis Establishes the Role of MRD Negativity in Long-Term Survival Outcomes in Patients With Multiple Myeloma. Blood Adv (2020) 4(23):5988–99. doi: 10.1182/bloodadvances.2020002827 PMC7724898 33284948

[79] Yong K Hinsley S De Tute RM Sherratt D Brown SR Flanagan L. Maintenance With Carfilzomib Following Carfilzomib, Cyclophosphamide and Dexamethasone at First Relapse or Primary Refractory Multiple Myeloma (MM) on the Phase 2 Muk Five Study: Effect on Minimal Residual Disease. Blood (2018) 132(Supplement 1):802–2. doi: 10.1182/blood-2018-99-116426

[80] Paiva B Chandia M Puig N Vidriales MB Perez JJ Lopez-Corral L. The Prognostic Value of Multiparameter Flow Cytometry Minimal Residual Disease Assessment in Relapsed Multiple Myeloma. Haematologica (2015) 100(2):e53–5. doi: 10.3324/haematol.2014.115162 PMC4803143 25381128

[81] Avet-Loiseau H San-Miguel JF Casneuf T Iida S Lonial S Usmani SZ. Evaluation of Sustained Minimal Residual Disease (MRD) Negativity in Relapsed/Refractory Multiple Myeloma (RRMM) Patients (Pts) Treated With Daratumumab in Combination With Lenalidomide Plus Dexamethasone (D-Rd) or Bortezomib Plus Dexamethasone (D-Vd): Analysis of Pollux and Castor. Blood (2018) 132(Supplement 1):3272–2. doi: 10.1182/blood-2018-99-113177

[82] Avet-Loiseau H Sonneveld P Moreau P Offner F van der Velden VHJ Caillot D. 82 Daratumumab (DARA) With Bortezomib, Thalidomide, and Dexamethasone (VTd) in Transplant-Eligible Patients (Pts) With Newly Diagnosed Multiple Myeloma (NDMM): Analysis of Minimal Residual Disease (MRD) Negativity in Cassiopeia Part 1 and Part 2. Atlanta, GA, United States: ASH (2021).

[83] Raje N Berdeja J Lin Y Siegel D Jagannath S Madduri D. Anti-BCMA CAR T-Cell Therapy Bb2121 in Relapsed or Refractory Multiple Myeloma. N Engl J Med (2019) 380(18):1726–37. doi: 10.1056/NEJMoa1817226 PMC8202968 31042825

[84] Costa LJ Derman BA Bal S Sidana S Chhabra S Silbermann R. International Harmonization in Performing and Reporting Minimal Residual Disease Assessment in Multiple Myeloma Trials. Leukemia (2021) 35(1):18–30. doi: 10.1038/s41375-020-01012-4 32778736

[85] Diamond B Korde N Lesokhin AM Smith EL Shah U Mailankody S. Dynamics of Minimal Residual Disease in Patients With Multiple Myeloma on Continuous Lenalidomide Maintenance: A Single-Arm, Single-Centre, Phase 2 Trial. Lancet Haematol (2021) 8(6):e422–32. doi: 10.1016/S2352-3026(21)00130-7 34048681

[86] Kumar SK Harrison SJ Cavo M de la Rubia J Popat R Gasparetto C. Venetoclax or Placebo in Combination With Bortezomib and Dexamethasone in Patients With Relapsed or Refractory Multiple Myeloma (BELLINI): A Randomised, Double-Blind, Multicentre, Phase 3 Trial. Lancet Oncol (2020) 21(12):1630–42. doi: 10.1016/S1470-2045(20)30525-8 33129376

[87] Palumbo A Cavallo F Gay F Di Raimondo F Ben Yehuda D Petrucci MT. Autologous Transplantation and Maintenance Therapy in Multiple Myeloma. N Engl J Med (2014) 371(10):895–905. doi: 10.1056/NEJMoa1402888 25184862

[88] Gay F Oliva S Petrucci MT Conticello C Catalano L Corradini P. Chemotherapy Plus Lenalidomide Versus Autologous Transplantation, Followed by Lenalidomide Plus Prednisone Versus Lenalidomide Maintenance, in Patients With Multiple Myeloma: A Randomised, Multicentre, Phase 3 Trial. Lancet Oncol (2015) 16(16):1617–29. doi: 10.1016/S1470-2045(15)00389-7 26596670

[89] Gay F Cerrato C Petrucci MT Zambello R Gamberi B Ballanti S. Efficacy of Carfilzomib Lenalidomide Dexamethasone (KRd) With or Without Transplantation in Newly Diagnosed Myeloma According to Risk Status: Results From the FORTE Trial. J Clin Oncol (2019) 37(15_suppl):8002–2. doi: 10.1200/JCO.2019.37.15_suppl.8002

[90] Costa LJ Chhabra S Godby KN Medvedova E Cornell RF Hall AC. Daratumumab, Carfilzomib, Lenalidomide and Dexamethasone (Dara-KRd) Induction, Autologous Transplantation and Post-Transplant, Response-Adapted, Measurable Residual Disease (MRD)-Based Dara-Krd Consolidation in Patients With Newly Diagnosed Multiple Myeloma (NDMM). Blood (2019) 134(Supplement_1):860–0. doi: 10.1182/blood-2019-123170

[91] Landgren O Hultcrantz M Lesokhin AM Mailankody S Hassoun H Smith EL. Weekly Carfilzomib, Lenalidomide, Dexamethasone and Daratumumab (wKRd-D) Combination Therapy Provides Unprecedented MRD Negativity Rates in Newly Diagnosed Multiple Myeloma: A Clinical and Correlative Phase 2 Study. Blood (2019) 134(Supplement_1):862–2. doi: 10.1182/blood-2019-126378

